# Conductance through single biphenyl molecules: symmetric and asymmetric coupling to electrodes

**DOI:** 10.3762/bjnano.6.171

**Published:** 2015-08-04

**Authors:** Karthiga Kanthasamy, Herbert Pfnür

**Affiliations:** 1 Institut für Festkörperphysik, ATMOS, Leibniz Universität Hannover, Appelstr. 2, D-30167 Hannover, Germany; 2 Laboratorium für Nano- und Quantenengineering, Leibniz Universität Hannover, Schneiderberg 30, D-30167 Hannover, Germany

**Keywords:** biphenyl, metallic break junctions, molecular conductance

## Abstract

The contacts and the chemical bonds formed between metallic electrodes and molecules determine to a large extent the conductive properties of single molecular junctions, which represent the smallest possible active elements in an electronic circuit. We therefore investigated in a comparative study, using the break junction technique (MCBJ), the conductive properties of [1,1’-biphenyl]-4,4’-dithiol (**M1**) and of 4’-mercapto-[1,1’-biphenyl]-4-carbonitrile (**M2**) between gold electrodes. As a function of electrode separation, characterized by the conductance close to 0 V, we found several plateaus of relative stability, with those close to 0.01G_0_ being the most pronounced. The overall conductance of symmetric and asymmetric molecules were surprisingly similar, only the range of stability was smaller for **M2**. While **M1** yielded symmetric *I*–*V*-curves, only small asymmetries were detected for **M2**. These are also reflected in the comparable values for coupling parameters using the single level resonance model. The high conductance for the asymmetric molecule is interpreted as a result of coherent coupling of electronic states through the whole molecule, so that the outcome cannot be predicted just by adding conductive properties of individual molecular groups.

## Introduction

The ultimate goal of molecular electronics is to implement single molecules as active functional elements in future electronic devices such as amplifiers, rectifiers, diodes and logic switches [1]. Two decades after the proposal from Aviram and Ratner describing the molecular junction as p-n diodes [2] the experimental research in the field of molecular electronics [3] emerged. Even today, our understanding of the fundamental properties and charge transport mechanism through the molecule is still far from being complete, although significant progress has been made [4–6]. The main reasons are the complexity of the metal–molecule–metal junctions and their control on the atomic level.

Even in the simplest case with a single molecule and well defined atomic contacts, the junction consists of the metal electrodes, the molecule and, most important, the molecular endgroups that form the bonds with the electrodes. While typically electrodes consisting of noble [7–8] and transition metals like Pt and Pd [9–10] have been used experimentally, several different types of molecules have been characterized ranging from simple alkane chains [11–12] to rather complex molecules [13–14].

The end groups determine the strength of coupling in metal–molecule–metal junctions and influence the frontier energy orbitals. Several experiments have also been carried out with different end groups like amine, nitro, nitrile, carboxy, iso cyanate, pyridine. The electron donating group leads to higher conductance while electron withdrawing groups have a smaller conductance. The Au–S thiol is a well known anchoring group due strong covalent bond coupling [15]. The iso cyano group has been found to be a promising group with high stability and high surface mobility [16–20], but studies have also been carried out for amines (NH_2_), carboxyls (COOH), dimethylphosphines (PMe_2_) and selenols (SeH) [21–24]. Mostly the experiments have been carried out with symmetric molecules while only a few experiments [6,25–27] have been done with molecules with asymmetric end groups and contacts.

One technique widely used for studying transport through single or few molecules is the mechanically controllable break junction (MCBJ), which can be used in different environments like vacuum [28–29], or in liquids [30]. Extensions of this method by addition of a third electrode allow even for electrical gating on the nanoscale [31]. The use of tunneling microscopy [6] or atomic force microscopy or a combination of both are further interesting and partly complementary techniques [32]. We employ the MCBJ technique to contact the molecules because of its robustness and the high stability.

One of the prototype molecules investigated in the past both experimentally [21,32–34] as well as theoretically [35–39] is the biphenyl molecule with various end groups, because of its high stability, so that rectifying behavior as well as thermoelectric properties could be studied. In this paper, we concentrate on the variation of the end groups for this molecule. Starting with [1,1’-biphenyl]-4,4’-dithiol as a reference, we compare the conducting properties with those of the asymmetric molecule with one thiol and one carbonitrile end group. This choice is motivated by two effects: Firstly, the interaction between thiol and gold electrodes is stronger than for the carbonitrile group [19]. Secondly, coupled with the details of bond formation is the fact that these two end groups have an opposite effect on the level alignment of the highest occupied (HOMO) and lowest unoccupied (LUMO) molecular levels [19,40]. While thiol tends to pull the HOMO up by shifting a small amount of charge to the molecule, the cyano group has the opposite effect. These properties may also depend on the exact binding geometries. As a result for biphenyl molecules with symmetric end groups (i.e., dithiol- (BTP) and dicyanobiphenyl (BCP)) conduction was mainly through the HOMO for BTP and through the LUMO for BCP, which also had an order of magnitude lower conductance [19]. Here we show that there is clear coupling between end groups and interaction over the whole molecule, so that there is still fairly high conductance through this asymmetric biphenyl molecule.

## Experimental

**Figure 1:**
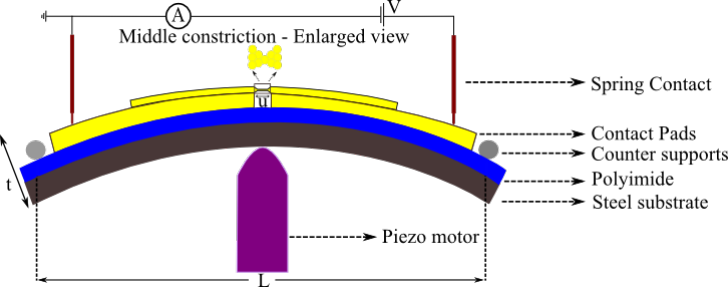
Schematic of our break junction setup.

A flexible stainless steel sheet (200 μm thick) coated with an insulating polyimide layer is used as a substrate. The gold electrodes are fabricated by an e-beam lithographic technique (for more details, see Supporting Information File 1). The sample is mounted on a homemade three point bending system and pumped down to high vacuum (10^−7^ mbar). A piezo motor was used to create strain in the center part of the constriction and break the junctions. The schematic setup is shown in Figure 1. The reduction factor, *r*, for our sample was calculated according to the geometry with formula 6*tu*/*L*^2^ [41] where *t* denotes the thickness of the substrate , *u* the distance between free standing bridges, and *L* the distance between counter supports. For our setup (*t* = 0.1378 mm, *u* = 2 μm, *L* = 15 mm), *r* is found to be 7.34 × 10^−6^.

**Figure 2:**
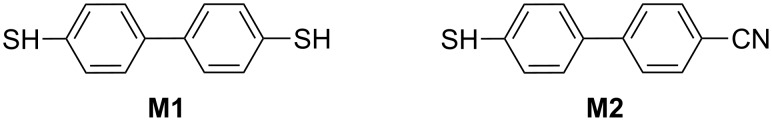
Chemical structures of [1,1’-biphenyl]-4,4’-dithiol (**M1**) and 4’-mercapto-[1,1’-biphenyl]-4-carbonitrile (**M2**).

The molecules were purchased from Sigma–Aldrich and dissolved to 1 mM concentration in toluene for [1,1’-biphenyl]-4,4’-dithiol (**M1**), and to 10 mM concentration for 4’-mercapto-[1,1’-biphenyl]-4-carbonitrile (**M2**) in toluene. The chemical structures are shown in Figure 2. Droplets of solutions of the molecules were put on the gold electrodes and dried before inserting them into vacuum. The same procedure was applied to gold thin films (100 nm) thermally evaporated onto silicon substrates, and the XPS signal (XPS *=* X-ray photoelectron spectroscopy) was measured in a UHV chamber after adsorption of molecules in the solvent for 24 h.

The current–voltage curves were measured with a Keithley 2401 sourcemeter, controlled by a LAB view program. We carried out two types of measurements: Firstly, the junctions were opened and closed many times using the piezo motor and the current was measured. The opening and closing cycles were used to construct a histogram. The most stable conformations of the molecule are seen as peaks in the conductance histogram. The junction after addition of molecules showed step-wise changes in the conductance of the molecule below 1G_0_. Secondly, we did series of *I*–*V*-measurements at the stable conductance values, as determined from the histograms.

## Results and Discussion

As shown by XPS (see Supporting Information File 1, Figures S2–S4), we get adsorption of both **M1** and **M2** molecules on the gold surfaces of the electrodes under the experimental conditions mentioned above. From comparison of Au4f with N2s and S2p signals, taking into account the atomic sensitivity factors, we get an estimate for thiol-bonded biphenyls that about one third of a monolayer is adsorbed after having the material in solution for 24 h. This agrees with a comparison of data collected during experiments with 4-hydroxythiophenol on Ag(100) [42]. Interestingly, the S2p signal of **M2** is smaller by a factor of six compared to **M1**, which means that the achievable **M2** concentration is lower by a factor of three, although the concentration in solution was ten times higher. With the MCBJ we use a few droplets of molecular solution. The solvent evaporated within one to two minutes in air. Therefore, the surface concentration will be much smaller and we expect only few molecules at the junction. The nitrogen peak from **M2** molecule confirms the presence of the molecule **M2** on the gold surface.

**Figure 3:**
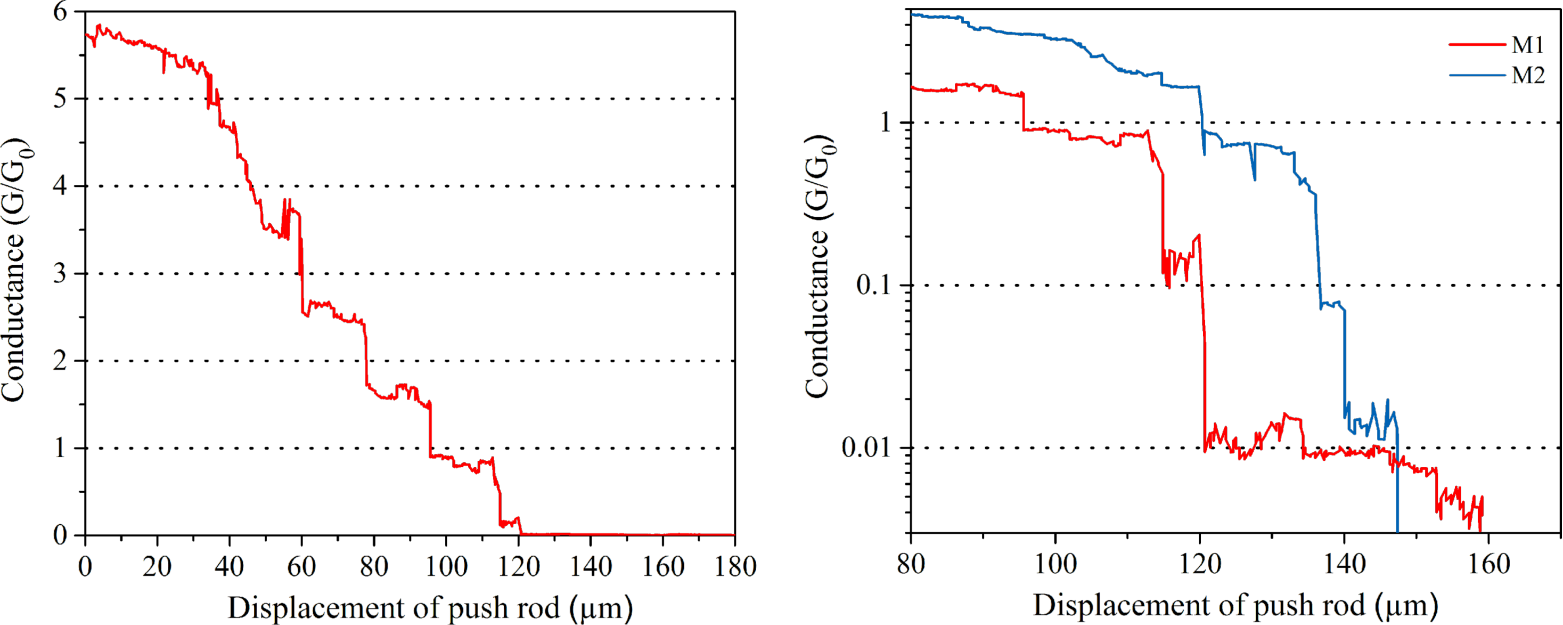
Typical curves of conductance versus mechanical displacement in presence of [1,1’-biphenyl]-4,4’-dithiol (**M1**, red) and 4’-mercapto-[1,1’-biphenyl]-4-carbonitrile molecules (**M2**, blue) during opening the break junction. Left: Conductance in presence of **M1** (linear scale), right: comparison of conductance in presence of **M1** and **M2** molecules.

As a first step of molecular conductance, we performed opening and closing cycles. In Figure 3 we show two characteristic curves of changes in conductance during opening the contacts in presence of **M1** and **M2** molecules. Conductance values close to and above 1G_0_ are obviously characteristic of direct metallic contacts between the electrodes, which presumably exist in parallel to molecular contacts. As can be seen from the left part of Figure 3, the conductance changes stepwise with the motion of the piston in the break junction. Assuming that these steps are due to the generation of a new (111)-oriented layer of gold on one of the contacts [43], the step distances in conductance on the x-axis coincide within 1% with the layer distance in this direction of gold (2.494 Å) taking into account the attenuation factor of the break junction given above. Since a conductance significantly below 1G_0_ is expected for single molecules [36], the steps seen in the right part seem to be mostly due to molecular properties. The symmetric **M1** molecule shows characteristic steps at 0.11G_0_, a very extended plateau at 0.011G_0_ and a gradual decrease for larger contact separations. It abruptly loses contact at a few times 10^−3^G_0_. The range of contact distances where molecular properties are seen, is typically 0.5 nm for **M1** and thus significantly smaller than the molecular size along its axis. While the conductance range at and above 1G_0_ may still be governed by direct metallic contacts, contacts at much smaller conductance must be formed by single (or few) molecules and are characteristic for bond formation by mercapto and nitrile end groups. This behavior is not unexpected, since the shape of break junctions is not necessarily regular, so that the direct metallic contact happens at a location different from the molecular contact. This effect is even more severe for the **M2** molecule, as seen by the blue curve in the same figure, which exhibits plateaus at similar conductance values, which, however, are significantly shorter. The shorter plateaus may be again a hint for the weaker bond formed by the nitrile end group compared to thiol.

**Figure 4:**
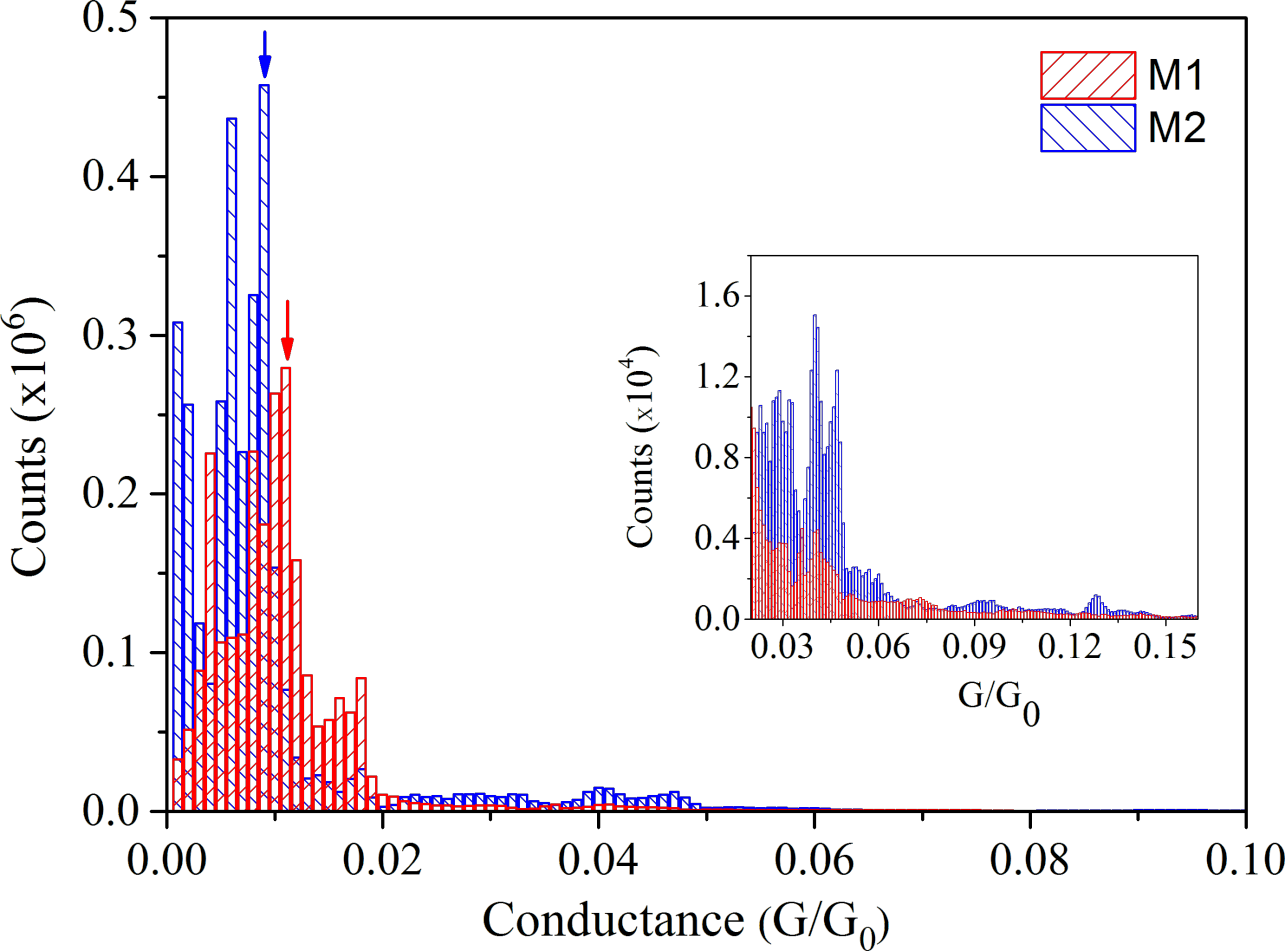
Conductance histograms determined for molecules **M1** and **M2** at +1 mV during opening and closing cycles of the break junction. Inset: Enlarged conductance regime above 0.02G_0_.

From Figure 3 it is clear that the length of the conductance plateaus is not always the same, presumably because of changes in bonding sites, or of molecular conformation between the electrodes for every opening and closing cycle. Therefore, we plot a 1D histogram taking into consideration all opening and closing cycles without any data selection. These histograms are shown for both molecules **M1** and **M2** in Figure 4. The frequency counts are done with a bin size of 0.001G_0_. The inset in Figure 4 shows the enlarged range of conductance above 0.02G_0_.

Both molecules show a pronounced maximum of conductance close to 0.01G_0_ (marked by arrows in Figure 4). The existence of multiple peaks may be explained by selective adsorption of sulfur atoms at steps sites or at flat gold surfaces, which results in large differences of conductance [15]. Conductance values above 0.02G_0_ have a weight of only 0.3% of all measured values, and are thus quite unlikely. Molecular conductance values for **M2** up to 0.16G_0_ were observed, whereas for **M1** this range goes only up to 0.04G_0_. In order to check for stable conformations of the molecules with respect to the electrodes, we performed several open and closing cycles. When the conductance was below 1G_0_ we operated the piezo motor with a constant stretching rate 0.15 pm/s. The conductance remained stable in a certain range of distances irrespective of stretching rate (up to few Å displacement), due to bond reformation or thermodynamic stability of the molecular junction [44–45] as already explained. We stopped at these particular positions and recorded multiple sweeps of current versus voltage. Some of these positions turned out to be stable over several hours.

**Figure 5:**
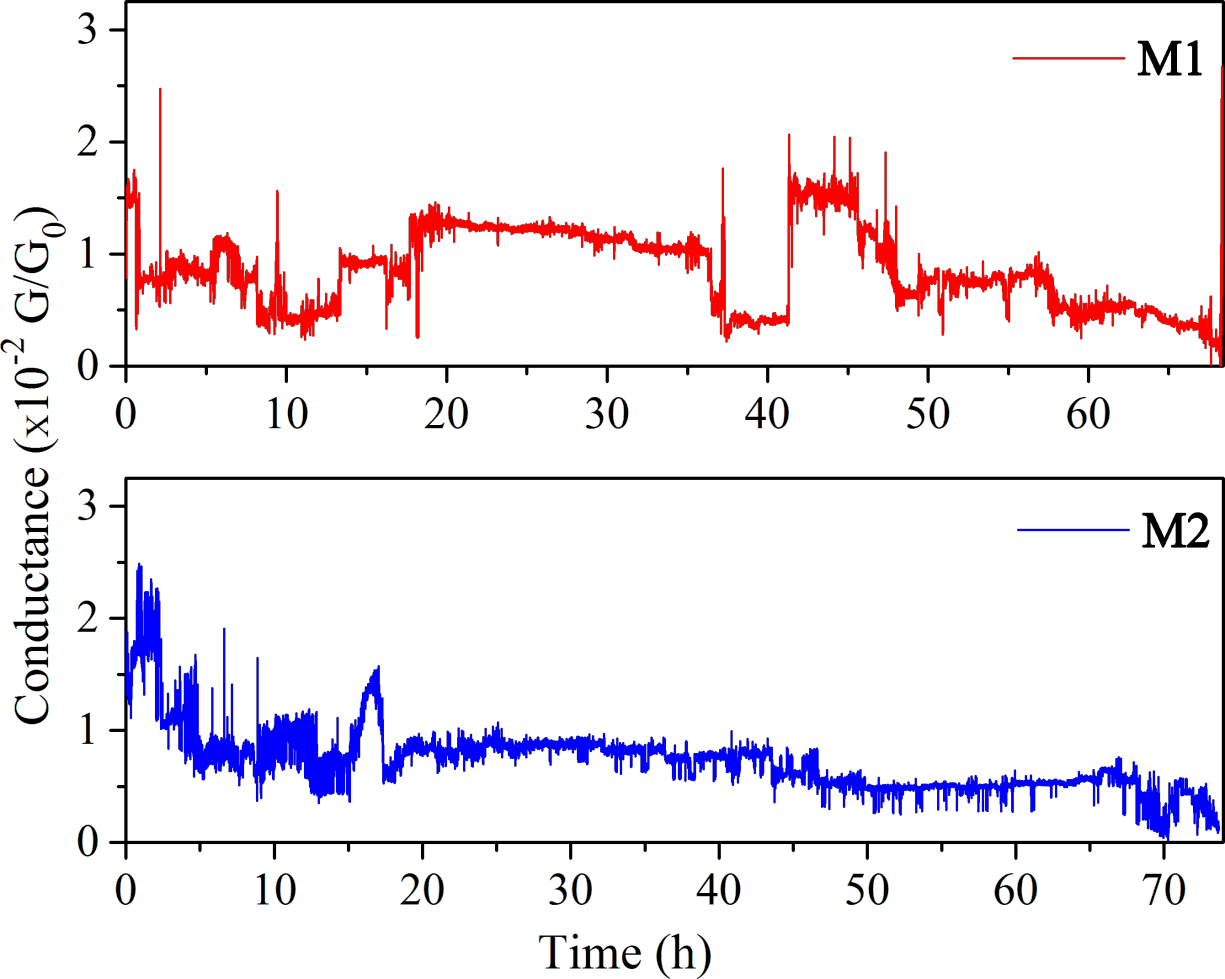
Conductance versus time at values close to the maxima of Figure 4 at room temperature for **M1** (upper panel) and **M2** (lower panel).

At such positions the conductance at constant 1 mV was monitored. Tests were performed at room temperature and at 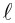
*N*_2_. No significant differences were found. As seen from Figure 5, particularly stable conductance of the molecules occurs close to the maxima of conductance shown in Figure 4. The same stable conductance conditions were obtained with several samples, which shows that a specific single molecular conformation is favorable giving rise to molecular conductance.

The conductance for the **M1** molecule was found to be stable around 0.01G_0_, but there are some fluctuations over time, possibly due to changes in bonding sites of the molecule on the electrodes, which have a great impact on conductance. The pronounced switching in conductance at times between 36–46 h has been characterized earlier as blinking of the thiol–gold bond, a characteristic of any molecule bonded to gold surface via thiol bonding [46]. A similar effect is to be expected by changes of the tilt angle between the phenyl rings and electrodes during bond fluctuations [35–36] as well as by spontaneous binding and unbinding of the molecular anchor groups to the electrodes [47]. A similar signature of the fluctuation was observed in the terphenyl dithiol molecule [48]. For the **M2** molecule, the fluctuation is small, possibly due to additional stability originating from the nitrogen bond to undercoordinated Au atoms [20]. In a recent experiment using electromigrated break junctions with gold electrodes [32] the stable conductance value obtained for the **M1** molecule is of the same order of magnitude as our values.

**Figure 6:**
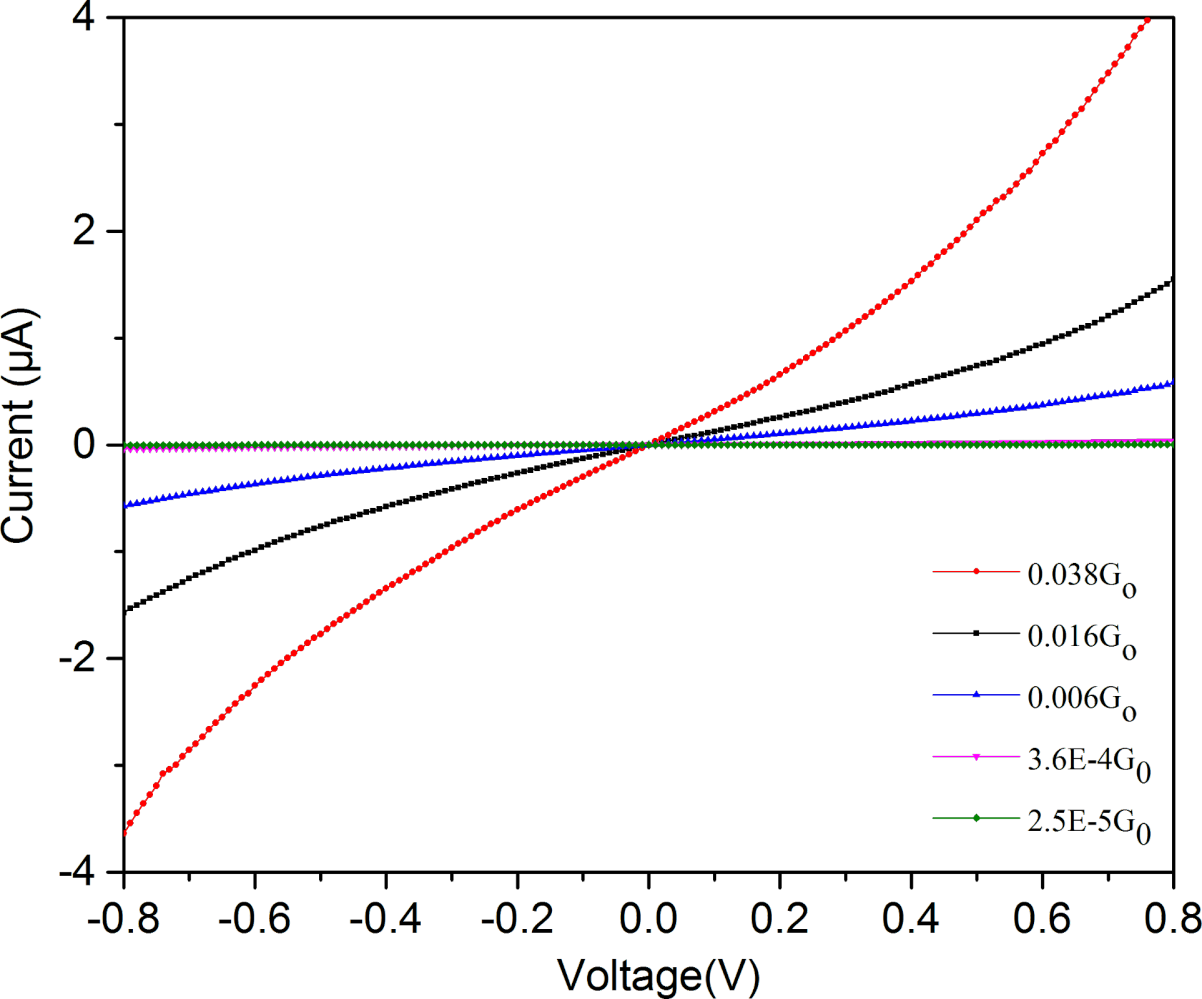
*I*–*V*-curves for the **M1** molecule at the conductance values indicated (measured close to 0 V at room temperature).

In the following we investigate the *I*–*V*-properties of both molecules in order to get more insight into the electronic conductive properties of the molecules, for which the electronic positions of HOMO and LUMO orbitals are of major importance. Only those measurements were selected in which conductance turned out to be stable (at small voltage) over at least several minutes. The value of conductance close to zero voltage is taken as measure for a certain molecular configuration between the contacts, since this is the only reference in the varying atomic rearrangements after every open and close cycle [49]. Starting from these stable reference conditions, the electrode distance could be changed to other still stable values of conductance, and measurements were done. At least 30 to 50 *I*–*V*-curves were measured, at each inter electrode distance and averaged. Some sample average *I*–*V*-curves are shown in Figure 6.

For the symmetric biphenyl molecule with thiol end groups we started our measurements at a conductance of 0.04G_0_ and investigated the conductance range down to 2 × 10^−4^G_0_, a range where we are quite sure to only investigate molecular conductance properties. The **M2** molecule turned out to be stable only down to 10^−3^G_0_, but measurements were possible up to 0.16G_0_, as seen in Figure 4. As expected, the *I*–*V*-curves for the **M1** molecule are fully symmetric and S-shaped [19]. Interestingly, asymmetric *I*–*V*-curves have been observed even for such symmetric molecules with an STM-based technique [50]. This finding may be due to the inherent asymmetric contact geometry and different chemical environment.

In order to get more quantitative insight, we used the single level resonance model, based on the Landauer formula, as described in [19]. Here the current *I*(*V*) is given by a transmission function *T*(*E*, *V*) through the molecule including contacts between the electrodes, characterized by Fermi functions *f* for a given voltage *V* between electrodes and given temperature

[1]
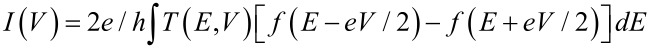


With the assumption that a single molecular orbital with energy *E*_0_ governs electronic transmission, the transmission function is given by the Breit–Wigner formula,

[2]
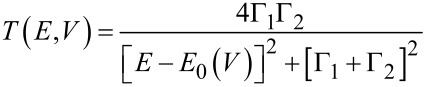


**Figure 7:**
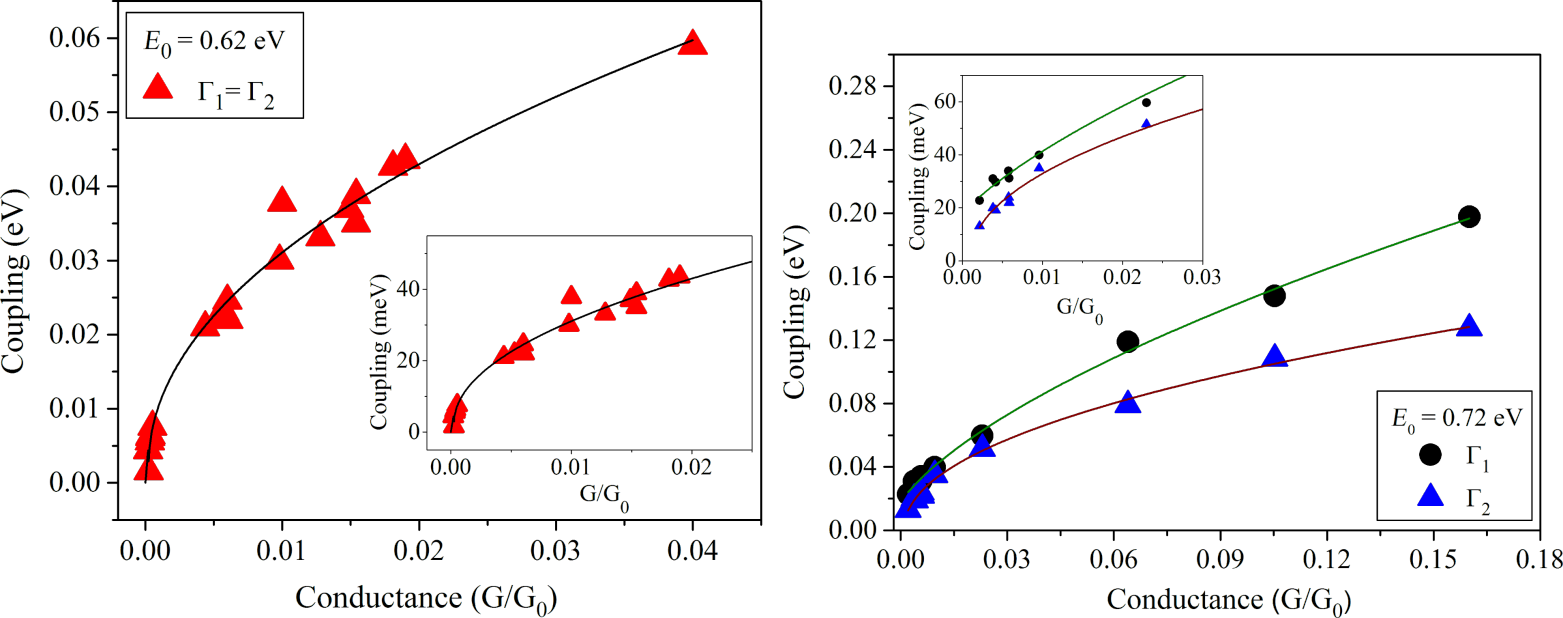
Coupling parameters as a function of 0 V-conductance extracted from the single level model fit for the symmetric **M1** molecule (left panel), and for the asymmetric **M2** (right panel). The lines drawn correspond to a square root dependence of the coupling parameters on conductance.

For the symmetric molecule, the coupling parameters to both sides are equal: Γ = Γ_1_ = Γ_2_. Using the single level model, Γ and *E*_0_ were varied in order to obtain a best fit to all measured *I*–*V*-curves. Model fits are shown in Supporting Information File 1. All *I*–*V*-curves could be fitted with a constant value for *E*_0_ = 0.62 ± 0.02 eV, irrespective of the conductance at small voltages. This value is found to be in agreement with the transition voltage measured with transition voltage spectroscopy (TVS) for **M1** [50]. The coupling strength, Γ, on the other hand, turned out to increase strongly and in a non-linear manner as a function of the conductance close to 0 V, with some scatter in the order of ±10%. The results are shown in Figure 7. In case of the asymmetric molecule (**M2**) the coupling parameters become unequal, Γ_1_ ≠ Γ_2_, and *E*_0_ depends on the Fermi level alignment with respect to left and the right electrodes according to

[3]
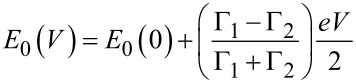


**Figure 8:**
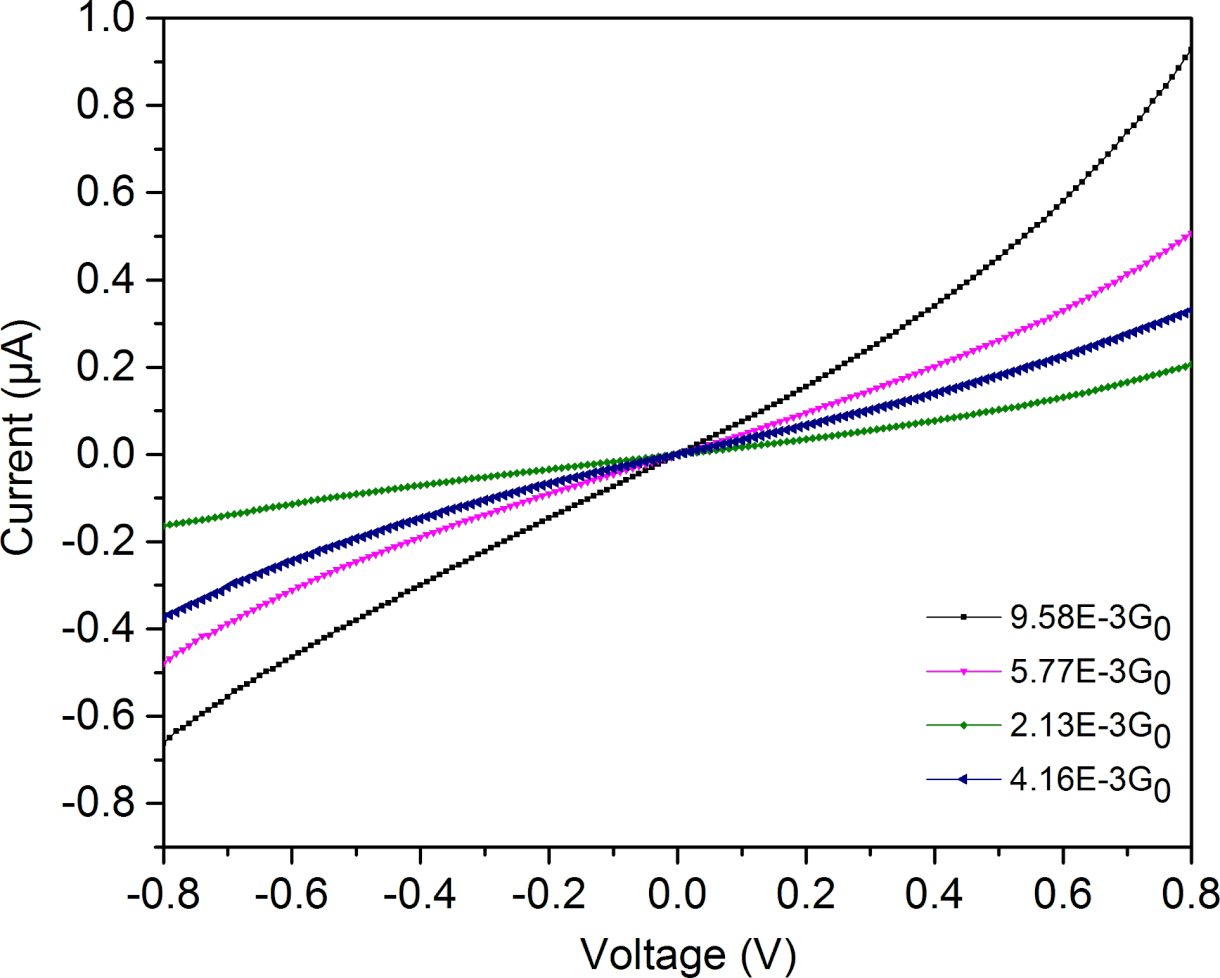
*I*–*V*-curves for the **M2** molecule at the conductance values indicated (measured close to 0 V) at room temperature.

For the **M2** molecule, the *I*–*V*-curves are shown in Figure 8, where only a slight asymmetry can be seen between positive and negative voltages. A similar data treatment was performed for molecule **M2**, but now with different coupling parameters Γ_1_ and Γ_2_. Again, a single value for *E*_0_ = 0.72 eV and varying values for the coupling parameters as a function of zero voltage conductance allowed a good fit to the data. The results are again summarized in Figure 7. As expected from the small asymmetries seen in Figure 8, the difference in the two coupling parameters is only few mV, which was also observed in the asymmetric Au–NH_2_–B–SH–Au molecular junction [6].

According to Equation 1, for small voltages a square root dependence of the coupling Γ on conductance can derived , if *E*_0_ is kept constant. This relation has been tested by fitting the coupling parameters for **M1** and **M2** using the equation

[4]
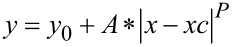


Indeed *P* = 0.5 ± 0.05 gives a rather good fit for **M1** and for **M2** with Γ_1_ ≠ Γ_2_. This demonstrates that the assumption of a constant *E*_0_ is reasonable in both cases within the single resonance model. For the symmetric dithiol end groups we note that there is fair agreement with data from other break junction measurements [32], both with respect to coupling constants and positions of effective resonance levels, with even somewhat higher conductance values for biphenyl and triphenyldithiol investigated in [19,48]. This contrasts with results of investigations carried out in electrolytes with tunneling tips [6], where conductance values and coupling parameters much smaller than ours have been obtained, although the positions of the resonant level obtained from fits are quite similar to the ones we obtained. While part of the deviations may originate from the number of investigated molecules being different, this cannot explain differences by orders of magnitude. Strong sensitivity of conductance to the bonding geometry is known from theoretical investigations [19–20,35–36,51], with variations in the observed range. On the other hand, it is unclear why such metastable configurations leading to very small conductance should be favoured in a certain type of experiment. These questions are still open.

The systematic increase of coupling parameters as a function of conductance close to zero voltage may have a similar origin. Although conductance was varied by almost two orders of magnitude, the positions of the resonance levels remain virtually unchanged for both types of end groups. This shows that the molecules stay intact and will also be deformed very little while the main character of the metal–molecule bonds, characterized by, e.g., the electron transfer, cannot be altered. Therefore, changes of bond angles [20,38], perhaps coupled with configurational changes at the metal electrodes, seem to be the most likely reason.

Perhaps the most surprising result of this study is the high conductance and the small asymmetry of the asymmetric molecule with thiol and carbonitrile end groups, when compared with predictions of pronounced rectifying behavior for the case of well separated binding groups in long alkane chains [40], and compared with results for the symmetric biphenyl with thiol and carbonitrile end groups [19]. Although we cannot exclude the presence of several molecules oriented in opposite directions in the junction, which would reduce the asymmetry, the high conductance remains. We interpret it as a signature for coherent coupling of electronic states through the whole molecule, which is responsible for this finding, very much in contrast to an incoherent addition of resistors.

## Conclusion

Our investigation of single molecules of [1,1’-biphenyl]-4,4’-dithiol in comparison with 4’-mercapto-[1,1’-biphenyl]-4-carbonitrile, using the break junction technique, shows high conductance for both molecules with only slight asymmetries in the *I*–*V*-curves for the asymmetric molecule. While the results for biphenyl dithiol are in fair agreement with those carried out previously in dry MCBJs, the contact formation seems to be significantly different in other experiments, as, e.g., with an STM-BJ. The reasons are still unclear. The high conductance for the asymmetric molecule is interpreted as a result of coherent coupling of electronic states through the whole molecule, so that the outcome cannot be predicted just by adding conductive properties of individual molecular groups.

## Supporting Information

File 1 Supporting information features details of the setup, XPS data, and details of the data evaluation.
