# Supplementary material for: Conductance through single biphenyl molecules: symmetric and asymmetric coupling to electrodes
Source: Beilstein J Nanotechnol. 2015 Aug 4;6:1690–7. doi: 10.3762/bjnano.6.171 (PMC4578413; doi:10.3762/bjnano.6.171)
Supplement: File 1 — Supporting information features details of the setup, XPS data, and details of the data evaluation. [file Beilstein_J_Nanotechnol-06-1690-s001.pdf]

Supporting Information

for

Conductance through single biphenyl molecules: symmetric and asymmetric coupling to electrodes

Karthiga Kanthasamy[1] and Herbert Pfnür*[1,2]

Address: [1]Institut für Festkörperphysik, ATMOS, Leibniz Universität Hannover, Appelstr. 2, D-30167 Hannover, Germany and [2]Laboratorium für Nano- und Quantenengineering, Leibniz Universität Hannover, Schneiderberg 30, D-30167 Hannover, Germany

Email: Karthiga Kanthasamy - karthiga@fkp.uni-hannover.de, Herbert Pfnür - pfnuer@fkp.uni-hannover.de

* Corresponding author

More experimental details

Sample Fabrication

The flexible stainless steel substrate was cleaned by ultrasonication in isopropanol and acetone for 10 minutes. The adhesion promoter (VM-651) and the PI-2610 (HD,Microsystems) (2 μm) were spin coated onto the stainless substrate and cured according to the procedure described elsewhere [1]. PI-2610 acts as an electrical insulating layer. The contact pads (Cr-8 nm and Au-100 nm) are deposited using a shadow mask evaporation technique. We then spin coated the samples sequentially with the positive resists methyl metha acrylate (MMA) and poly methyl methyl acrylate (PMMA) (200 nm and 180 nm, respectively) as a double layer. They were baked at 150 °C (MMA) and at 180 °C (PMMA) for 90 s, respectively. The structures with a middle constriction of 150 $\times$ 300 nm were written at 30 kV by e-beam lithographic technique using a JEOL SEM 5900 instrument. Later the samples were developed using MIBK/isopropanol (1:3) solution for 50 s and were then immersed into isopropanol for 15 s to stop the development. Cr (8 nm) and Au (70 nm) were deposited onto the sample by electron beam and thermal evaporation, respectively. The substrate was then soaked in acetone and heated to 60 °C for 20 min to remove the resist. Reactive ion etching (RIE) was done in an O_2/CHF_3 atmosphere with 80:20 ratio and a power of 30 watt for 4 min in a RIE Alcatel machine. The etching rate was found to be 85 nm per minute under these conditions, which was confirmed by AFM measurements. A scanning electron microscope (SEM) image of a typical gold electrode after fabrication is shown in Figure S1.

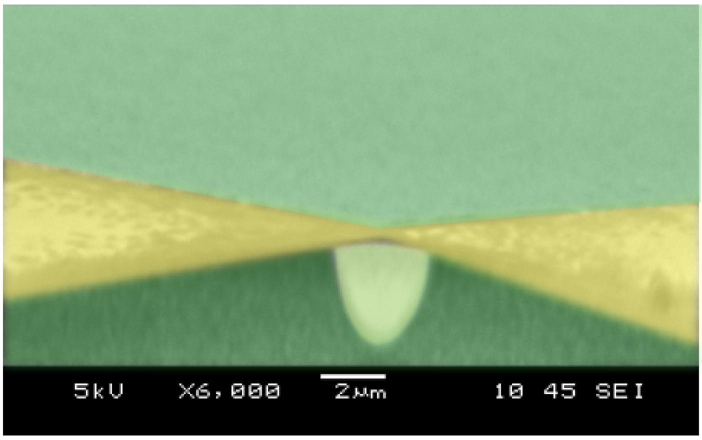

Figure S1: SEM image of MCBJ fabricated by electron beam lithography.

X-ray photoelectron spectroscopy (XPS)

The XPS technique was used in this experiment to identify the adsorption of the molecules on to the surface. The presence of S2p signal from 1 mM 4,4'-biphenyl dithiol (**M1**), and both S2p, N1s signals from 10 mM 4'-mercaptobiphenyl carbonitrile (**M2**) confirms the adsorption of these molecules on the gold surface. Systematic studies were done by XPS to find the optimum concentration of **M2** (starting from 1 mM). An adsorption time of 24 h and a concentration of 10 mM was found to be suitable for detection of XPS characteristic peaks.

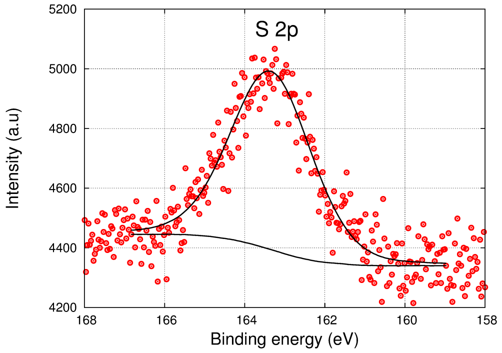

(a) S2p peak from **M1**.

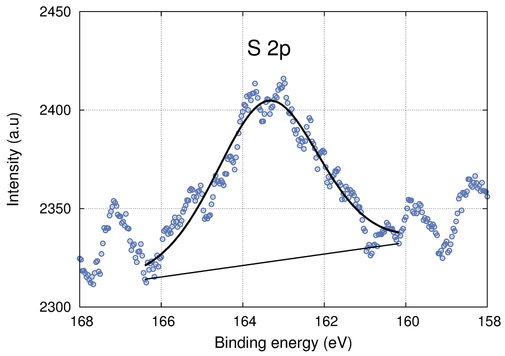

(b) S2p peak from **M2**.

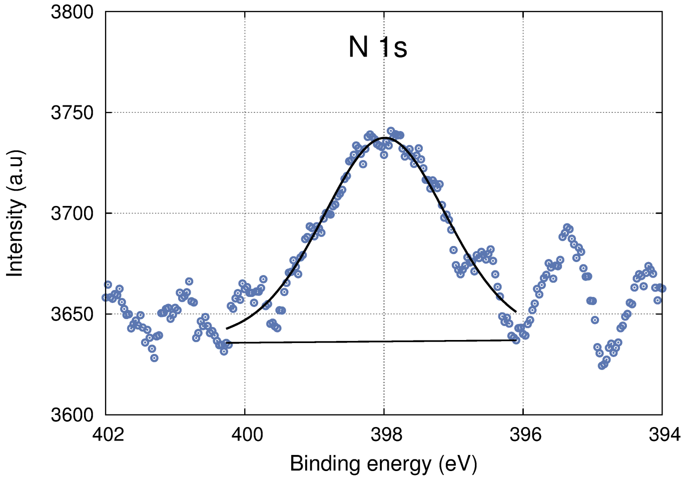

(c) N1s peak from **M2**.

Figure S2: XPS characteristic peaks.

The XPS characteristic peaks for the two molecules show that the molecules after adsorption stay stable on gold surface without any oxidation. Due to low signal from molecule **M2** no quantitative analysis is possible.

Single level model fit

The graphs shown in Figure S3, S4 are examples for single level fits of the experimental *I*–*V*-curves for molecules **M1** and **M2** using symmetric and asymmetric transmission functions to derive the current described in main manuscript. The values for E_0, Γ_1, Γ_2 obtained from the fits are plotted in Figures 8 and 9 in the main manuscript.

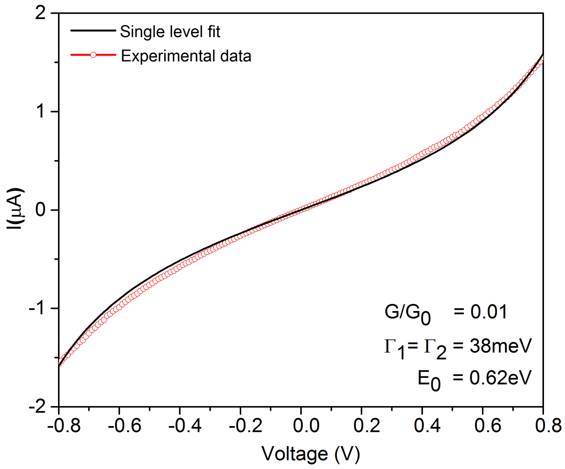

Figure S3: Single Level model fit with **M1** experimental *I*–*V*-curve.

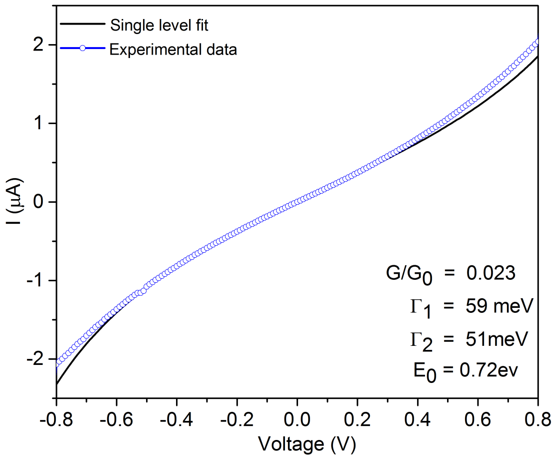

Figure S4: Single Level model fit with **M2** experimental *I*–*V*-curve.

The model fits perfectly the experimental curves for the bias region below ±0.4 V, whereas above this bias range there is deviation up to 10%, which may be due to an additional channel or to inelastic tunneling contributing to the electronic transport. Both contributions cannot be explained

within this model.

Reference:

1. HD Microsystems, Product Bulletin
